# Dyslipidemia: A Trigger for Coronary Heart Disease in Romanian Patients with Diabetes

**DOI:** 10.3390/metabo10050195

**Published:** 2020-05-14

**Authors:** Mihnea-Alexandru Găman, Matei-Alexandru Cozma, Elena-Codruța Dobrică, Nicolae Bacalbașa, Ovidiu Gabriel Bratu, Camelia Cristina Diaconu

**Affiliations:** 1 Faculty of Medicine, “Carol Davila” University of Medicine and Pharmacy, 8 Eroii Sanitari Boulevard, 050474 Bucharest, Romania; matei.cozma@gmail.com (M.-A.C.); codrutadobrica@gmail.com (E.-C.D.); nicolae.bacalbasa@umfcd.ro (N.B.); ovi78doc@yahoo.com (O.G.B.); camelia.diaconu@umfcd.ro (C.C.D.); 2 Center of Hematology and Bone Marrow Transplantation, Fundeni Clinical Institute, 258 Fundeni Road, 022328 Bucharest, Romania; 3 Department of Visceral Surgery, Fundeni Clinical Institute, 258 Fundeni Road, 022328 Bucharest, Romania; 4 Urology Clinic, Carol Davila University Emergency Central Emergency Military Hospital, 88 Mircea Vulcanescu Street, 010825 Bucharest, Romania; 5 Academy of Romanian Scientists, 54 Splaiul Independentei, 050085 Bucharest, Romania; 6 Internal Medicine Clinic, Clinical Emergency Hospital of Bucharest, 8 Calea Floreasca, 014461 Bucharest, Romania

**Keywords:** diabetes, dyslipidemia, coronary heart disease, ischemic heart disease, statins

## Abstract

Previous studies have reported age and gender disparities in the occurrence and therapeutic approach of dyslipidemia and (or) coronary heart disease (CHD) in patients with type 2 diabetes mellitus (T2DM). We aimed to investigate these differences in Romanian patients with T2DM. A cross-sectional, observational, retrospective study was conducted using the medical records of T2DM patients who attended the outpatient facility of the Internal Medicine Clinic of the Clinical Emergency Hospital of Bucharest, Romania for routine check-ups in a six-month period. We analyzed the records of 217 diabetic patients (mean age 69 ± 11 years; 51.15% women). We found no significant gender differences in the occurrence of dyslipidemia, CHD or CHD + dyslipidemia or in terms of statin prescription. However; patients aged 65 years or older were significantly more affected by dyslipidemia, CHD or CHD + dyslipidemia, versus subjects aged <65 years. Further, they were more likely to be prescribed statin therapy (*p* < 0.0001 for all). Statins were prescribed to 67.24% of the patients with dyslipidemia; 61.01% of the subjects with CHD; and to 91.48% of the patients who had both conditions. e recorded no gender differences in the occurrence of CHD and (or) dyslipidemia in Romanian T2DM patients. Patients aged 65 years or older had a higher prevalence of CHD and/or dyslipidemia, and were more likely to be prescribed statins, versus younger counterparts. However, many T2DM patients with CHD and (or) dyslipidemia were undertreated: Nearly 33% of the subjects with dyslipidemia, and nearly 40% of the ones with CHD were not prescribed statins.

## 1. Introduction

Type 2 diabetes mellitus (T2DM) is currently a major public health issue, not only in developing countries, but also worldwide. It is estimated that by the year 2030, the number of patients who will suffer from T2DM—most of whom will be aged 45–60 years—will increase by up to 70%, reaching a total of about 439 million [1]. While in previous generations, T2DM was diagnosed almost exclusively in elderly patients, due to the high prevalence of obesity, sedentary lifestyles and hyper-caloric diets in children, increasing numbers of adolescents and young adults are diagnosed nowadays with T2DM [1].

One of the most common causes of morbidity and mortality in T2DM is coronary heart disease (CHD). Coronary heart disease accounts for about 65% of the deaths of diabetic patients and shares numerous pathogenic links with T2DM, with many authors sustaining that inflammation is the common element between the two entities [2,3,4]. It has been shown not only that T2DM is an independent risk factor for CHD, but also that its association with other traditional risk factors, such as hypertension, smoking, obesity and a sedentary lifestyle, causes a more aggressive injury of the heart, leading in time to the rapid degradation of the cardiac function [5]. In patients with diabetes, chronic hyperglycemia plays an important role in the development of cardiovascular disease since it aggravates atherosclerosis [6].

A key contributor in the development of CHD is dyslipidemia [7]. Dyslipidemia is characterized by high plasma levels of triglycerides (TG), low HDL–cholesterol (HDLc) concentrations and increased concentrations of small dense LDL–cholesterol (LDLc) particles [5]. Of these three categories of plasma lipids, it has been shown that high levels of LDLc are the most related to cardiovascular risk. Moreover, estimates have shown that, in the United States, approximately 70% of patients with T2DM have LDLc levels >100 mg/dL and only 3% of the diabetic patients have successfully reached the latest targets recommended by the American Diabetes Association (ADA) for all three plasma lipids [3]. Besides a proper diet and an active lifestyle, there are pharmacological strategies to lower LDLc levels, such as statins, fibrates, ezetimibe, cholestyramine and torcetrapib, but by far the most employed ones are statins which lower cholesterol synthesis by inhibiting the 3-hydroxy-3-methylglutaryl coenzyme A (HMG–CoA) reductase. In addition to this effect, statins also have anti-inflammatory properties on the atherosclerotic plaque, lower plasma levels of acute phase proteins and overall reduce the risk of mortality and morbidity from cardiovascular disease. Commonly prescribed statins include atorvastatin, simvastatin, pravastatin and rosuvastatin [8].

Several studies have reported age and gender differences in T2DM regarding the occurrence of CHD and dyslipidemia [9,10,11]. Although women benefit from a relative cardiovascular protection given by the hormonal profile before menopause, it seems that this advantage is lost if they suffer from diabetes [11]. According to recent reports, in T2DM, the excess risk of CHD is 44% higher in women versus men [12]. Other studies state the contrary: Al-Zakwani et al. (2018) have reported a lower prevalence of CHD in diabetic women versus men (17% versus 40%, *p* < 0.001), but a higher prevalence of dyslipidemia. However, they found out that men were more likely to be prescribed statins and to achieve lipid goals versus women [13]. Taking this information into account, our aim was to investigate age and gender disparities in the occurrence of CHD and dyslipidemia in diabetic patients, as well as age and gender disparities in the prescription of statins.

## 2. Results

Our study group involved 217 diabetic patients (mean age 69 ± 11 years; 51.15% women). In terms of dyslipidemia and CHD occurrence, we recorded the following (Table 1):

➢ A total of 58 patients (58/217, 26.72%) only had dyslipidemia: 30 women (30/58, 51.73%) and 28 men (28/58, 48.27%). Although we observed a tendency for women to have dyslipidemia, there was no statistical significance for this finding (*p* = 1.00). In terms of age, 32 patients had <65 years (32/58, 55.17%) and 26 were aged ≥65 years old (26/58, 44.83%) (mean age = 56.60 ± 7.26 vs. 74.14 ± 6.94 years, *p* < 0.0001).
➢ A total of 59 patients (59/217, 27.18%) only had CHD: 30 women (30/59, 50.85%) and 29 men (29/59, 49.15%). Although we observed a tendency for women to have CHD, there was no statistical significance for this finding (*p* = 1.00). In terms of age, 16 patients had <65 years (16/59, 27.11%) and 43 were aged ≥65 years old (43/59, 72.89%) (mean age = 54.43 ± 8.66 vs. 76.44 ± 7.27 years, *p* < 0.0001).
➢ A total of 47 patients (47/217, 21.65%) had both dyslipidemia and CHD: 24 women (24/47, 51.06%) and 23 men (23/47, 48.94%). Although we observed a tendency for women to have both CHD and dyslipidemia, there was no statistical significance for this finding (*p* = 1.00). In terms of age, 13 patients had <65 years (13/47, 27.65%) and 34 were aged ≥65 years old (34/47, 72.35%) (mean age = 59.45 ± 3.88 vs. 73.31 ± 6.54 years, *p* < 0.0001).
➢ Other comorbidities reported in our study group were obesity in 73 patients (73/217; 33.64%), hypertension in 174 patients (174/217; 80.18%), chronic heart failure in 105 patients (105/217; 48.38%), chronic kidney disease in 84 patients (84/217; 38.70%), atrial Fibrillation in 94 patients (94/217; 43.33%), diabetic nephropathy in 25 patients (25/217; 11.52%), diabetic neuropathy in 22 patients (22/217; 10.13%) and peripheral arterial disease in 28 patients (28/217; 12.90%).

**Figure 1:**
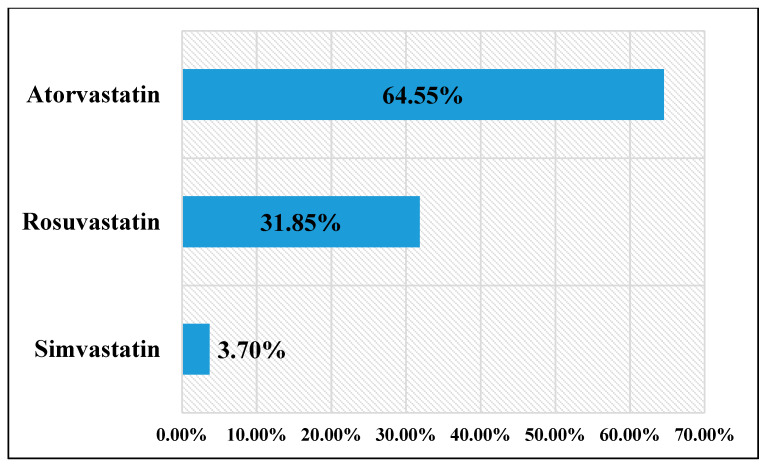
Types of statins administered in our study group.

A total of 135 patients (135/217, 62.21%) were prescribed statin therapy: 63 women (63/135, 46.67%) and 72 men (72/135, 53.33%). Patients aged <65 years were less likely to receive statins versus patients aged 65 years or more (mean age = 56.72 ± 6.93 vs. 74.27 ± 6.71 years, *p* < 0.0001). There was a tendency for men to receive statins in a greater fashion, but there was no statistical significance for this finding (*p* = 0.09). Prescription patterns (depicted in Figure 1) were: atorvastatin–87 patients (87/135, 64.45%), rosuvastatin—43 patients (43/135, 31.85%) and simvastatin—5 patients (5/135, 3.70%). Patients diagnosed only with dyslipidemia received statins in 39 cases (39/58, 67.24%). CHD patients were given statins in 36 cases (36/59, 61.01%). Patients suffering from both CHD and dyslipidemia received statins in 43 cases (43/47, 91.48%). A number of 17 patients (17/53, 32.07%) without CHD or dyslipidemia received statins for the prevention of cardiovascular events.

**Figure 2:**
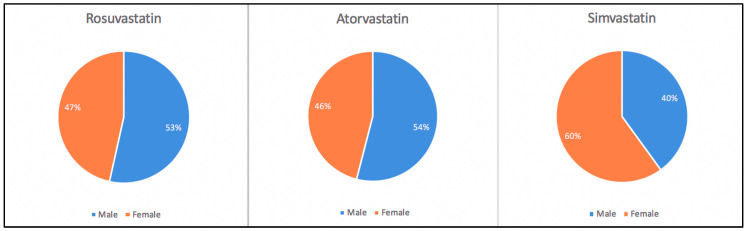
Prescription patterns of statins by gender.

Atorvastatin was prescribed in 87 patients: 47 men (47/87, 54.02%) and 40 women (40/87, 45.97%). Rosuvastatin was prescribed in 43 patients: 23 men (23/43, 53.48%) and 20 women (20/43, 46.51%). Simvastatin was prescribed only in 5 cases: 2 men (2/5, 40.00%) and 3 women (3/5, 60.00%). There were no significant differences in terms of gender regarding the prescription of these drugs (*p* > 0.05) (Figure 2).

In terms of diabetes management, the patients were prescribed:

➢ Oral antidiabetic agents in 107 cases (107/217, 49.31%);
➢ Insulin in 29 cases (29/217, 13.36%);
➢ Oral antidiabetic agents + insulin in 26 cases (26/2017, 11.98%);
➢ Lifestyle recommendations only (diet, physical exercise) in 55 cases (55/217, 25.35%).

In terms of other medications, the patients were prescribed beta-blockers (153/217, 70.51%), antiplatelet agents (112/217, 51.61%), angiotensin converting enzyme inhibitors (103/217, 47.47%), calcium channel blockers (98/217, 45.16%), anticoagulants (86/217, 39.63%), angiotensin–receptor blockers (46/217, 21.20%) or nitrates (37/217, 17.05%).

## 3. Discussion

Our study examined the occurrence of dyslipidemia and CHD in Romanian patients with T2DM, aiming to identify possible age and (or) gender differences in subjects who associated these comorbidities. Diabetic women had a tendency to suffer more from dyslipidemia, CHD or dyslipidemia + CHD versus their male counterparts, but these findings lacked statistical significance (*p* > 0.05). However, subjects aged ≥65 years had a higher prevalence of dyslipidemia, CHD or dyslipidemia + CHD versus their younger (<65 years) counterparts (*p* < 0.0001 for all). Thus, we may hypothesize that age plays an important role in the development of dyslipidemia and (or) CHD in Romanian patients with T2DM. Other studies have reported, however, gender differences in the occurrence of dyslipidemia and (or) CHD in patients with T2DM. For example, Al-Zakwani et al. (2018) studied 3336 patients with diabetes and reported a higher prevalence of CHD in men versus women (40% versus 17%, *p* < 0.001). The prevalence of the metabolic syndrome and the lipid levels during statin therapy were, however, higher in women versus men [13]. Other studies, however, have reported that diabetic men and women suffer from dyslipidemia in a similar proportion (34.2% vs. 35.3%, *p* = 0.369) [14]. Billimek et al. (2015) showed that females diagnosed with T2DM are less likely to achieve target goals for lipids, and also had a higher prevalence of dyslipidemia versus men [15]. Anto et al. (2019) reported that dyslipidemia is more prevalent in diabetic males rather than females and that subjects aged 40 or elder are particularly affected by lipid disturbances [16]. In terms of CHD prevalence, Li et al. (2017) described an 18.1% prevalence of CHD in diabetic males and 10.4% in diabetic females, and that CHD is found in 19.6% of the subjects aged ≥65 years versus 10.3% in the subjects aged 18 to 64 years. In addition, they support the hypothesis that CHD is associated with the age and the gender of T2DM patients, concluding also that male sex and advanced age were particularly associated with the presence of CHD in diabetic subjects [17].

In terms of statin prescription, older subjects (aged ≥65 years) were more likely to be prescribed lipid-lowering therapy versus younger counterparts (aged < 65 years). We also observed a tendency for men to be prescribed statin therapy versus women, but this finding lacked statistical significance (*p* > 0.05). The most commonly administered statins were atorvastatin and rosuvastatin and, in a smaller number, simvastatin, with no gender preference associated with their prescription. Santalucia et al. (2015) reported that patients aged ≥65 years are frequently subjected to polypharmacy and that elder men are more likely to receive statin therapy versus women both at hospital admission (22.9% vs 18.3%; *p* = 0.0008), as well as on discharge from the hospital (25.2% vs 19.6%; *p* < 0.0001) [18]. Gender differences in statin prescription have also been confirmed in a large scale study conducted in the United States: Nanna et al. (2019) analyzed the records of 5693 subjects eligible for prescription of lipid-lowering medication and concluded that males were more likely to receive statin prescriptions (78.4% versus 67.0%) or lipid-lowering therapy at the intensity suggested by the available guidelines (45.2% versus 36.7%) (*p* < 0.001 for both). In addition, in some cases, 18.6% of females versus 13.5% of males reported not having been proposed to undergo statin therapy at any time during their examinations [19]. Thus, urgent measures are needed to lower gender disparities in the management of cardiovascular risk in diabetic patients, and particularly in women, taking into consideration that the presence of T2DM seems to reduce the premenopausal hormonal protection in females [20].

Interestingly, however, many patients with dyslipidemia or (and) CHD were undertreated. Patients with dyslipidemia received statins in 67.24% of the cases, whereas subjects who suffered from CHD were prescribed lipid-lowering drugs only in 61.01% of the instances. Patients who had both dyslipidemia and CHD received statins in a better proportion, 91.48%. Cardiovascular prevention was the motif of lipid-lowering drugs’ prescription in 32.07% of the patients enrolled in our study. The decision not to initiate statin therapy may be based on the results of several studies which failed to demonstrate the benefit of statin therapy in the primary prevention of cardiovascular events in patients with T2DM. The West of Scotland Coronary Prevention Study (WOSCOPS) and Air Force/The Texas Coronary Atherosclerosis Prevention Study (AFCAPS/TexCAPS) did not enroll a sufficient number of patients, while the Antihypertensive and Lipid-Lowering Treatment to Prevent Heart Attack Trial (ALLHAT), which had a group of 10,355 patients, of whom 35% (3638 subjects) had T2DM, showed a 9% reduction in the CR after treatment with pravastatin 40 mg, which proved to be not statistically significant. The mean LDLc in the pravastatin-treated group decreased by an average of 23 mg/dL [3,21,22,23,24].

Currently, the American Diabetes Association has several recommendations for statin therapy initiation in patients with T2DM. Therefore, patients with T2DM and CVD or at risk of developing CVD >20% at 10 years, regardless of age, should start high-intensity statin therapy. Patients with T2DM who are under 40 years old and have other cardiovascular risk factors should begin moderate statin therapy along with lifestyle changes. In addition, patients with T2DM and CVD who have LDLc >70 mg/dL and are already receiving the maximum tolerated statin dose may benefit by taking another lipid-lowering drug, but with another mechanism of action (e.g., ezetimibe) [25]. Irrespective of the currently available armamentarium in the management of T2DM and related conditions, lifestyle adjustments and the primary prevention of T2DM, CHD or dyslipidemia remain of uttermost importance [26]. Lifestyle interventions remain, however, difficult to implement, even in subjects who are aware of the risks of an unhealthy diet and of sedentarism, such as medical students and physicians [27,28,29].

According to the PREDATORR (PREvalence of DiAbeTes mellitus, prediabetes, overweight, Obesity, dyslipidemia, hyperuricemia and chronic kidney disease in Romania) study, dating back from 2016, the prevalence of T2DM in the Romanian population was estimated at 11.6% (95% confidence intervals: 9.6%–13.6%). Also, the PREDATORR study found a high prevalence of dyslipidemia in Romanian diabetic patients: 65.4% had high LDLc levels, 43.8% had low HDLc levels and 47.0% suffered from hypertriglyceridemia. Interestingly, the aforementioned study found in the univariate analysis significant associations between low HDLc (odds ratio: 1.8, 95% confidence intervals: 1.4–2.3, *p* < 0.001) and high TG (odds ratio: 2.2, 95% confidence intervals: 1.8–2.8, *p* < 0.001) and the presence of T2DM, but the odds of having known T2DM were lower in patients with high LDLc levels (odds ratio: 0.4, 95% confidence intervals: 0.3–0.5, *p* < 0.001). The multivariate logistic regression also confirmed these results: high TG levels (odds ratio: 1.7, 95% confidence intervals: 1.2–2.3, *p* < 0.01) were predictors of known T2DM, whereas high LDLc levels (odds ratio: 0.4, 95% confidence intervals: 0.3–0.5, *p* < 0.001) were associated with a lower prevalence of known T2DM. At that time, the authors argued that the latter was due to aggressive statin interventions in diabetic patients with high LDLc levels [30]. However, our study reported an under-prescription of statins in T2DM patients with dyslipidemia or CHD, with only T2DM subjects who associated both these comorbidities being prescribed statins in an appropriate fashion to reduce their cardiovascular risk. Irrespective of the local approach, it must not be forgotten that in T2DM a high percentage of deaths is still linked to cardiovascular disorders and particularly to an increased burden of atherosclerosis. Dyslipidemia remains a modifiable cardiovascular risk factor for whose management we possess a great variety of pharmacological agents, not only statins, but the efforts to tackle atherogenic lipid abnormalities in T2DM still disappoint. Considering dyslipidemia can affect nearly 85% of subjects with T2DM, urgent measures are needed to reduce its prevalence in diabetes, some of which can include changes in lifestyle to decrease the burden of obesity, improvements in cardiovascular risk stratification, treatment of lipid profile abnormalities (with statins, ezetimibe, bile acid sequestrants, fibrates as most appropriate) in combination with glucose-lowering agents and targeting other modifiable cardiovascular risk factors, and an accurate assessment of adherence and efficiency of therapy [31,32]. Moreover, comorbidity clusters heavily impact the management and quality of life of patients with T2DM and definitely lead to a worse prognosis of the disease. A recent Canadian study assessing the prevalence of comorbidities and comorbidity clusters in 448 736 community-dwelling T2DM elderly subjects concluded that the most common comorbidities in T2DM are hypertension (79.1%), arthritis (59.6%), other cardiovascular disorders (59.3%), CHD (37.6%), anxiety (36.9%) and dyslipidemia (33.7%). Only 30,949 T2DM subjects had one single comorbidity, with dyslipidemia ranking the 4th (*n* = 2031, 6.6%) and CHD the 7th (*n* = 1067, 3.4%) in terms in prevalence in this subgroup of patients. In the subgroup of T2DM subjects who had two comorbidities (*n* = 56,700), dyslipidemia was frequently associated with hypertension (3rd most common combination, *n* = 5699, 10.0%) or arthritis (10th most common combination, *n* = 1609, 2.85%), whereas CHD most commonly coexisted with hypertension (7th most common combination, *n* = 2193, 3.9%). In the 74 314 diabetic patients who had ≥3 comorbidities, dyslipidemia often was diagnosed in association with arthritis and hypertension (2nd most common combination, *n* = 5305, 7.1%), hypertension + other cardiovascular conditions (5th most common combination, *n* = 2672, 3.6%) or anxiety + hypertension (10th most common combination, *n* = 1959, 2.6%). CHD was most commonly encountered in combination with hypertension + other cardiovascular conditions (4th most common combination, *n* = 3738, 5.0%) or arthritis + hypertension (9th most common combination, *n* = 2111, 2.8%) [33]. Non-modifiable risk factors, such as genetic traits, are unquestionably involved in the development of T2DM and related comorbidities. In their investigation of genetic factors influencing the development of diabetes and CHD, Osman et al. (2020) found out that all patients who suffered from CHD were also diabetic and that the most frequent association variant for CHD was the rs264 single nucleotide polymorphisms in the LPL gene, a gene which encodes the lipoprotein lipase (odds ratio for allele A: 1.96, *p* = 0.009). The rs1977833 single nucleotide polymorphisms in the HHEX gene was strongly associated with T2DM (odds ratio for allele A: 0.56, *p* = 0.0016), whereas HDLc and TG levels were associated with the BRAF and ZEB2 genes, respectively [34].

### The Dyslipidemia–Inflammation–Diabetes–Cardiovascular Disease Pathway

Recent advances in the field of metabolomics have improved our current knowledge regarding the development of T2DM and its related complications. The discovery of novel biomarkers of disease has contributed to our understanding of the dyslipidemia–inflammation–T2DM–cardiovascular disease pathway. For example, elevated levels of TG, aromatic or branched-chained amino acids and their derivatives, bile acids and lysophosphatidylcholine have been linked to an increased risk of T2DM. Other molecules, namely 1,5-anhydroglucitol and β-hydroxypyruvate, have been associated with mechanisms responsible for the control of glucose levels, whereas 1,5-anhydroglucitol, glutamic acid, glutamine, L-aspartic acid, norvaline, symmetric dimethylarginine and tyrosine have emerged as biomarkers which can predict the development of T2DM-related complications [35]. Other studies have also hypothesized that the prevalence of T2DM may be associated with essential and non-essential amino acids. Lu et al. (2019) have demonstrated that tyrosine and glutamine, two non-essential amino acids and isoleucine, leucine and valine, three essential amino acids, are all associated both with the incidence and prevalence of T2DM. Other essential amino acids, such as lysine and phenylalanine, have only been linked with the prevalence of T2DM, together with the alanine, glutamic acid and glycine, three non-essential amino acids. The essential amino acid tryptophan was only associated with the incidence of T2DM. Of all the aforementioned amino acids, however, only tyrosine and valine predicted T2DM independently [36]. Guasch-Ferré et al. (2016) reported, however, that only valine, leucine, isoleucine, tyrosine and phenylalanine were positively associated with T2DM risk, whereas for glutamine and glycine the association was negative [37].

Moreover, oxidative stress and inflammation have emerged as putative mechanisms in the development of cardiovascular disease in T2DM. Reactive oxygen species, via different metabolites and biomarkers, increase the activity of thrombocytes and lead to endothelial inflammation and proliferation of cells, as well as decrease the homeostasis in the vessel wall. For example, arachidonic acid, protein-kinase C, cyclooxygenases 1 and 2, prostaglandins G_1_ and H_1_ and peroxynitrite are related to an increase cardiovascular disease burden in T2DM via hyperactivation of platelets and reduction of endothelial homeostasis. Oxidized LDLc, p65/nuclear factor-kβ, monocyte chemo-attractant protein-1, several cytokines and growth factors (interleukins 1β, 6, 8 and 23, tumor necrosis factor-α, angiotensin II, interferon-1) are responsible for endothelial inflammation and cell proliferation, thus leading to the development of cardiovascular disease in T2DM [38]. Moreover, the adipokine leptin has inflammation, oxidative stress, atherogenesis and thrombosis-inducing properties and has been associated with CHD, T2DM and its related complications via the aforementioned mechanisms [38]. The gut microbiome has also been incriminated as a key player in the development of cardiometabolic disorders, such as atherosclerosis, obesity, metabolic syndrome, insulin resistance, T2DM, myocardial infarction, heart failure, hypertension and stroke [39,40,41]. Moreover, the risk of T2DM also related to metabolites of plasma vitamin D: Zheng et al. (2019) discovered a negative association between T2DM and non-epimeric 25-hydroxy-vitamin D3, whereas for epimeric 25-hydroxy-vitamin D3 the relationship was positive [42]. Lipid biomarkers, such as lipoprotein(a), have also been correlated with the risk of cardiovascular death (mainly acute myocardial infarction) and all-cause mortality [43].

Our study has, however, several limitations. First, it was designed as an observational–retrospective study. Second, we could not assess the patients’ adherence to the prescribed treatment, which would have consisted in a great addition to the manuscript. Thus, further research is needed to fill these knowledge gaps regarding diabetic patients with CHD and (or) dyslipidemia from Romania.

## 4. Materials and Methods

### 4.1. Study Design

We performed a cross-sectional, observational–retrospective review study of the electronic health records of diabetic patients who attended the outpatient facility of the Internal Medicine Clinic of the Clinical Emergency Hospital of Bucharest, a university-affiliated tertiary hospital in Bucharest, Romania. The Clinical Emergency Hospital of Bucharest is one of the largest university hospitals in Bucharest, serving as a referral center for patients living in Bucharest, the capital of Romania, as well as patients from other regions of our country.

### 4.2. Data Source and Data Extraction

Data were extracted from the electronic medical records of the Internal Medicine Clinic of the Clinical Emergency Hospital of Bucharest for a 6-month period in 2019. The data that were derived from the electronic medical charts included demographics (age, gender), clinical data and clinical diagnosis (T2DM and other comorbidities) and information related to the prescriptions given to patients on discharge from the hospital (drug names).

### 4.3. Ethics Approval and Consent to Participate

The study was approved by the Ethics Council of the Clinical Emergency Hospital of Bucharest, Bucharest, Romania (approval number: 4263/13.05.2019) and written informed consent was collected from all patients involved. All regulations imposed by the national law and the Declaration of Helsinki (1975), as revised in 2008(5), were thoroughly respected.

### 4.4. Study Population

Inclusion criteria: Adults aged ≥ 18 years of age, diagnosed with T2DM (using the ICD-10-CM clinical diagnoses codes), who attended the outpatient facility for routine check-ups during the study period. Exclusion criteria: Adults diagnosed with other conditions other than T2DM, diabetic patients whose charts lacked data about demographics, clinical data and prescription information, patients who were unable to provide informed consent upon hospitalization or who refused to partake in the study.

### 4.5. Statistics

We presented categorical variables as frequencies and percentages and continuous variables as the mean ± standard deviation (SD). Categorical variables were compared using Fisher’s exact test. Continuous variables were compared using independent samples t-test. The level of significance was presented as *p*-values. The analysis was performed at a 5% level of significance using GraphPad QuickCalcs (https://www.graphpad.com), MedCalc (https://www.medcalc.org) and Microsoft Excel (Microsoft Office Professional Plus 2013).

## 5. Conclusions

Our study did not find significant gender differences in the occurrence of CHD and (or) dyslipidemia in patients with T2DM. T2DM subjects aged ≥65 years had a higher prevalence of dyslipidemia, CHD or dyslipidemia + CHD versus their younger (<65 years) counterparts and were also more likely to receive statins. However, many patients with dyslipidemia or (and) CHD were undertreated: patients with dyslipidemia received statins in 67.24%, CHD subjects in 61.01% and patients who had both conditions in 91.48% of the cases, respectively. Further studies are needed to elucidate age and gender disparities in T2DM patients with dyslipidemia and CHD, both in terms of prevalence and therapeutic approach in the real-life setting.

## Figures and Tables

**Table 1:** Characteristics of the study group in terms of age, sex, presence/absence of CHD and/or dyslipidemia.

Variable	All	Women	Men
Age (years)	69 ± 11	71 ± 11	67 ± 10
Number	217 (100%)	111 (51.2%)	106 (48.8%)
Dyslipidemia	58 (26.72%)	30 (51.73%)	28 (48.27%)
CHD	59 (27.18%)	30 (50.85%)	29 (49.15%)
Dyslipidemia + CHD	47 (21.65%)	24 (51.06%)	23(48.94%)
None	53 (24.44%)	27 (50.94%)	26 (49.06%)
